# In search of the spectral composition of an effective light trap for the mushroom pest *Lycoriella ingenua* (Diptera: Sciaridae)

**DOI:** 10.1038/s41598-021-92230-y

**Published:** 2021-06-17

**Authors:** Sándor Kecskeméti, András Geösel, József Fail, Ádám Egri

**Affiliations:** 1 Department of Zoology, Plant Protection Institute, Centre for Agricultural Research, Eötvös Loránd Research Network, Budapest, Hungary; 2 Department of Vegetable and Mushroom Growing, Institute of Horticultural Science, Hungarian University of Agriculture and Life Sciences, Villányi út 29-43, Budapest, 1118 Hungary; 3 Department of Entomology, Institute of Plant Protection, Hungarian University of Agriculture and Life Sciences, Villányi út 29-43, Budapest, 1118 Hungary; 4 Institute of Aquatic Ecology, Centre for Ecological Research, Karolina út 29, Budapest, 1113 Hungary

**Keywords:** Animal behaviour, Animal physiology, Entomology

## Abstract

Certain fungus gnats, like *Lycoriella ingenua* are notorious pests in agriculture, especially in mushroom production. While larvae cause mainly direct crop damage, adults are vectors of several dangerous fungal pathogens. To promote the development of pesticide-free management methods, such as light trapping, we measured the spectral sensitivity of *L. ingenua* compound eyes with electroretinography and performed two different behavioural experiments to reveal the wavelength dependence of phototaxis in this species. The spectral sensitivity of the compound eyes is bimodal with peaks at 370 nm (UV) and 526 nm (green). Behavioural experiments showed that attraction to light as a function of wavelength depends on light intensity. In our first experiment, where the minimal photon flux (10^5^–10^9^ photons/cm^2^/s) needed for eliciting a phototactic response was determined wavelength by wavelength, phototaxis was strongest in the green spectral range (~526 nm). In the other behavioural experiment, where wavelength preference was tested under a higher but constant light intensity (~10^13^ photons/cm^2^/s), the highest attraction was elicited by UV wavelengths (398 nm). Our results suggest that both UV and green are important spectral regions for *L. ingenua* thus we recommend to use both UV (~370-398 nm) and green (~526 nm) for trapping these insects.

## Introduction

Members of the fungus gnat family Sciaridae (Diptera) are widespread around the globe, even at the most extreme places, such as subantarctic islands, tundras, and mountainous regions above 4000 m altitude^1,2^. Some species live exclusively in caves and others inhabit arid deserts^3^, but the most common habitat of sciarids are forests, swampy regions and moist meadows^4^. They live under tree barks of deadwood, decaying leaves, manure piles, and most commonly, in the soil^1^. They feed on soil-dwelling fungi and on the root hairs of plants, but they also consume decaying plant material and other organic matter as well^5^. Sciarids are small (size = 1–6 mm) dark-coloured winged insects having well developed compound eyes. On their ovoid head the compound eyes meet above the base of the antennae, forming a dorsal bridge. The antennae are fairly long, consisting of 14 flagellomeres and the *scapus* and *pedicellus* are rounded. Three ocelli are also present on the head. The palpus have usually three segments^6^. The available information regarding fungus gnat eye morphology is scarce, what we know is that the compound eye has relatively low, only a few hundred facets and the facet size is species dependent^7^. In general, most sciarids are not considered to be pests, but some species may be deemed to be harmful for agriculture^4^.

However, the presence of sciarids in mushroom production is far more serious, as they are considered to be the most important pests in this specialized crop production^8–10^. The most common species in mushroom are *Lycoriella castanescens, Lycoriella ingenua* and *Bradysia ocellaris*^9^. The economical threshold for fungus gnats is low, one larva in 125 g casing material may result in 0.5% crop loss^11,12^. Direct damage is caused by the larvae, which feed on the substrate (compost) of edible mushrooms^2^ by taking away nutrients and water from the crop itself^12^. The sphorophore primordias and fruiting bodies may also be damaged by larval tunnelling^13^, in addition, the vegetative hyphae are also threatened. Furthermore, the excrement of the larvae may shift the pH levels of the compost to a critical level, when it will no longer be suitable for the mycelial growth of the mushroom^14^. The most commonly damaged crop is white button mushroom, which derives from the volume of production of this fungus species. Apart from button mushroom, oyster, king oyster, shiitake, shimeji and to an extent almost every cultivation can be hindered by sciarid infestation^15,16^. The adults do not damage the mushroom directly, but can cause serious losses to farmers by transmitting fungal pathogens to the crop^2^. It has been stated, that individuals of the fungus gnat species *L. ingenua* are attracted by the volatilome of *Trichoderma* sp., which is one of the most severe fungal disease of button mushroom production, and the adult may serve as an important vector^17^. The adult can also carry the spores of *Lecanicillium fungicola* on its body, and contribute to the spread of bacterial blotch diseases^13^.

Crop protection of edible mushrooms against fungus gnats is difficult as the number of approved pesticides is low, or for some cultures, such as *Pleurotus* sp., there are no approved pesticides^18^. The application of entomopathogenic nematodes like *Steinernema feltiae* offers a reasonable control agent against gnats in button mushroom production^9,19,20^, but in the case of species like *Lentinula* sp., *Pleurotus* sp., *Cyclocybe* sp., where the compost is entirely covered in plastic foil before fruiting body formation, the reapplication of nematodes is problematic. Since the European Commission wishes to reduce the use of pesticides by 50% by 2030^21^, it is not plausible to expect a new insecticide to appear for farmers in mushroom production, at least in Europe.

Development of alternative pest management methods are key to the production of safer crops for consumption and to the reduction of the environmental load of pesticides. An important source of information for insects is the visual environment. For example, colour, polarization, and contrast are important visual cues available for the majority of animals^22^. The most common method against insect pests is the attraction of individuals with light traps, however, trap efficiency strongly depends on the spectrum of the light^23^. The effectiveness of UV and green wavelengths in insect pest management has been demonstrated by several studies, and the application of LED light sources is becoming more and more frequent^23^. Besides trapping, there are further options, for example, presentation of light of a properly chosen intensity and wavelength can inhibit certain pests from entering a growing facility^24^. Another method is light adaptation, where the activity of nocturnal insects (e.g. mating, foraging etc.) are disrupted by the application of light sources during the night^23^.

One of the first studies about the spectral specific responses of sciarids to light was performed by Stringer and Meyer-Rochow^25^. Their results indicated that certain cave-dweller sciarids were mostly attracted by blue-green wavelengths, although the studied species was not identified. Later Jess and Bingham^26^ examined the responses of adult *L. ingenua* to different light source types. They stated that *L. ingenua* were positively phototactic even at low light levels, and the 300–650 nm spectral range attracted greater number of individuals than wavelengths above 700 nm. The positive phototaxis of fungus gnats was also verified by Cloyd et al.^27^, who found that specimens of *Bradysia* sp. nr. *coprophila* responded to light stimuli having intensity lower than 0.08374 µmol/m^2^/s (= 5.04·10^12^ photons/cm^2^/s). The first attempt for revealing the wavelength dependence of phototaxis of fungus gnats was made by Kim et al.^28^. By using a blue, green, orange and a red quasi monochromatic LED light trap they found that the green LED (528 nm) was the most attractive to *L. ingenua.* The potency of LEDs against *Bradysia difformis* fungus gnats were further explored by Stukenberg et al.^29^ who used 13 LEDs ranging from UV (371 nm) to amber (619 nm). Their results showed that the highest number of sciarids were attracted by UV (382 nm) and green-yellow wavelengths (532–592 nm) and their combination. They also used different backgrounds to verify the effect of contrast, and stated that a black surface was the most effective. The importance of contrast produced by a dark background for the chive gnat *Bradysia odoriphaga* has also been confirmed^30^. The very recent molecular identification of two UV sensitive and one long-wavelength sensitive opsin genes in *B. odoriphaga* (NCBI GenBank records at https://www.ncbi.nlm.nih.gov/genbank: MH491829.1; MK766510.1; MK766508.1) seems to be supporting the previously mentioned high attractiveness of UV and green wavelengths.

In this study we revealed the wavelength dependence of phototaxis of *L. ingenua* in two different behavioural experiments. Furthermore, we measured the spectral sensitivity of *L. ingenua* compound eyes with electroretinography because no electroretinogram of any sciarids has been published as far as we know. Studying the visual ecology of such pest insects may lead to the enhancement of light traps, and efficient light traps may help mushroom growers to reduce sciarid infestations on farms without the intense use of insecticides but rather through the mass trapping of the target organism.

## Materials and methods

### Fungus gnats

For laboratory experiments, we used *L. ingenua* adults, originating from pure insect cultures that were maintained as described by Kühne and Heller^31^. The colonies were initiated by collecting mating pairs of fungus gnats from a mushroom farm in Ócsa (47° 19′ 29″ N, 19° 18′ 27″ E). After mating, inseminated females were isolated and males were identified by examining their hypopygium^3,32^. Mated females of *L. ingenua* were placed in plastic breeding pots (870 ml) containing peat-moss (Kekillä DSM 3 W, Kekillä Professional, Vantaa, Finland) with approx. 95% water content and oat flakes with dried baker’s yeast granulate were provided as sustenance for the larvae. The pots were covered with plastic-fiber veil and were stored in an environmental control chamber in 16:8 L:D cycle, at 23 °C and 85% relative humidity.

### Electroretinography measurements

*L. ingenua* were placed with protruding head and thorax into a properly cut plastic pipette tip. Thorax, antennae and mouthparts were fixed to the pipette tip with melted paraffin wax and finally a wax droplet was placed between the thorax and head to make the head totally immobilized. For recording, tungsten microelectrodes (diameter = 0.08 mm) etched in saturated KNO_2_ solution were used. The recording electrode was inserted into the left eye to which the light stimulus was primarily delivered. The reference electrode was inserted to the other eye with its tip reaching the centre of the head.

A custom-built, calibrated light source containing 14 quasi monochromatic LEDs was used for stimulation of the eye preparations. Peak wavelengths (± half bandwidth) of the available LEDs were 346 nm (± 5.0 nm), 376 nm (± 4.8 nm), 402 nm (± 5.5 nm), 421 nm (± 6.4 nm), 442 nm (± 8.5 nm), 467 nm (± 10.4 nm), 496 nm (± 13.5 nm), 516 nm (± 14.5 nm), 552 nm (± 17.7 nm), 598 nm (± 6.9 nm), 623 nm (± 7.7 nm), 641 nm (± 8.6 nm), 660 nm (± 8.2 nm) and 744 nm (± 10.3 nm). The light intensity of the stimuli was variable through multiple log intensity units depending on the wavelength. Detailed description of the light source is described elsewhere^33^, where it was used in a behavioural experiment with a four-branch quartz light guide (Moritex SOHC4S3.5-1500S). For delivering light stimuli to the eye preparations, only one terminal of the light guide was used. The light beam reaching the eye preparation was 5 mm wide being approximately 16 times wider than the compound eye itself.

Receptor responses were amplified with a custom-built amplifier (see electronic circuit diagram in Supplementary Figure S1) inspired by Land et al.^34^. Data acquisition was done with a stereo USB sound card based on the C-Media CM6206 USB Audio I/O Controller chip (C-Media Electronics Inc., Taipei, Taiwan) using Audacity 2.2.1 audio recording software. The sound card was modified for low frequency and DC measurements as described on the homepage of Data AcQuisition And Real-Time Analysis (http://www.daqarta.com). Left and right channels of the sound card were used for recording the photoreceptor cell responses and a 450 mV reference signal, respectively, to a 16-bit WAV audio file with 8000 Hz sampling frequency. The reference signal facilitated data evaluation by indicating the presence of light stimulus and was also used for calibrating the voltage level of the measured signal on the left channel. In preliminary tests the linearity of the sound card inputs was verified in the ± 1 V range, hence the amplification was set to keep the measured signal within this region.

After 40 min of dark adaptation, electrodes were inserted under 660 nm light and a preprogrammed stimulus sequence was presented to the eye preparation after an additional 10 min of darkness. For each of the 14 available wavelengths, in increasing order, 6–8 500-ms-long light stimuli were applied with increasing light intensity with 3-s-long inter-stimulus intervals. Inter-stimulus interval of 12 s was used when wavelength was switched to the next one. This whole stimulus sequence was repeated *N* = 6–8 times with 1-min-long separating intervals. Recordings were made under 23–26 °C temperature, 50–55% relative humidity during the day when the behavioural experiments were typically performed. Photon flux of the applied light stimuli varied between 2.4·10^11^ and 2.4·10^15^ photons/cm^2^/s.

For a given preparation, based on the reference signal and the preprogrammed stimulus sequence, the responses were cropped from the recorded WAV file. Response amplitudes were considered as the magnitude of negative jumps in the potential in the first 100 ms of the 500-ms-long light stimulus (Fig. 4A). In other words, the lowest potential value in the first 100 ms of the stimulus relative to the potential measured at the beginning of the stimulus was registered as the response amplitude. For a given stimulus sequence, sigmoid exposure-response curves were fitted to the measured response amplitudes as a function of log photon flux for all available wavelengths. Spectral sensitivity was calculated by obtaining the reciprocals of photon flux required for inducing a standard response criterion for all wavelengths^35^. Finally, all spectral sensitivity curves corresponding to each repetition (*N *= 6–8) of the stimulus sequence were averaged and normalized with the maximal value. A total number of 12 female and 4 male *L. ingenua* eye preparations were measured.

### Behavioural experiment 1

Wavelength dependence of *L. ingenua* phototaxis was studied in two different experimental setups. In behavioural experiment 1, action spectrum of phototaxis was determined by measuring the critical light intensity eliciting a standard phototactic response criterion as a function of wavelength in the 368-637 nm spectral range.

**Figure 1:**
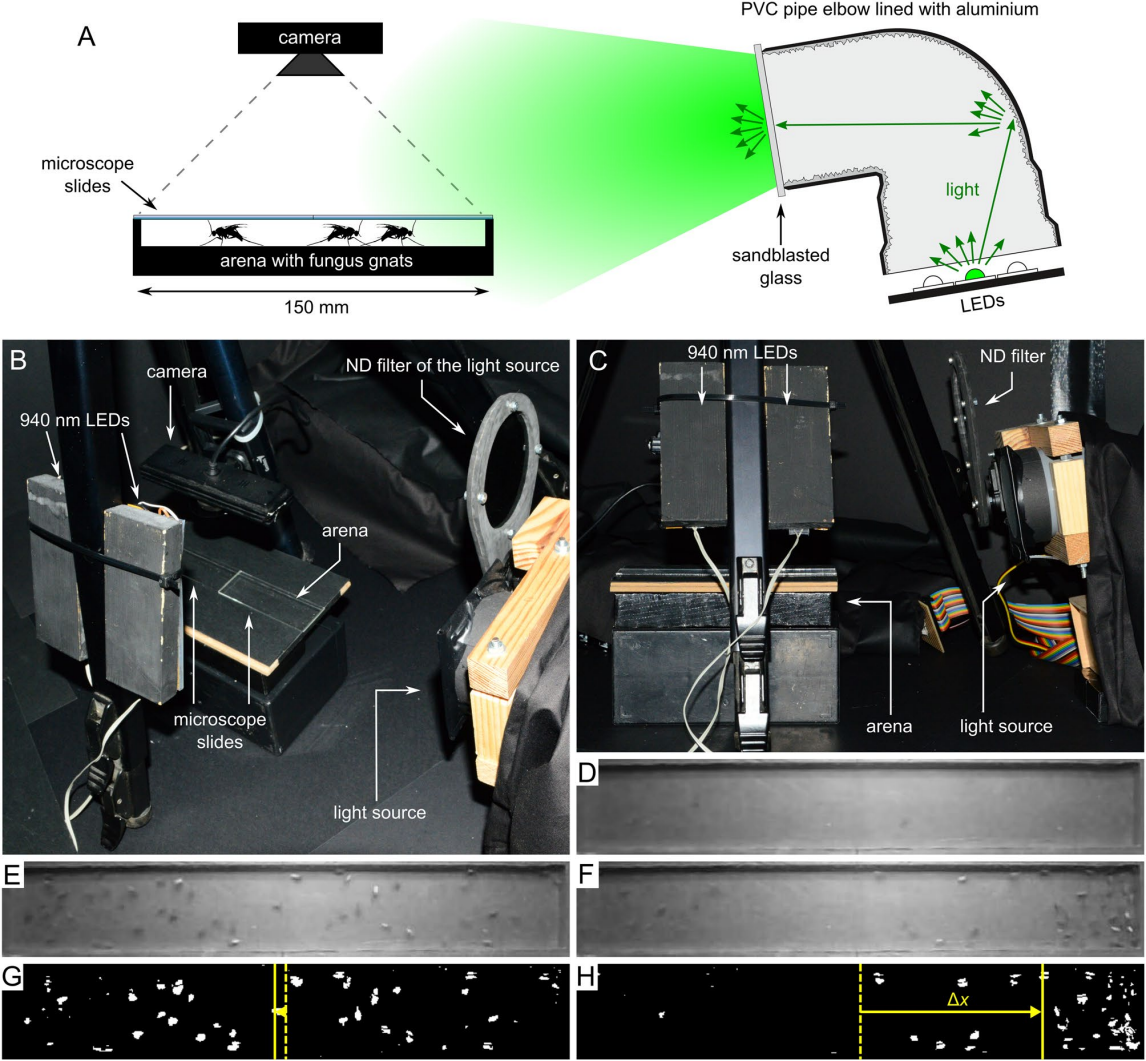
Experimental setup of behavioural experiment 1 and the evaluation process of the images. (**A**) Schematic diagram of the experimental setup. (**B**,**C**) Photograph of the setup. (**D**) Median image of the 10 photos corresponding to a stimulus (λ = 402 nm, photon flux = 3.61·10^8^ photons/cm^2^/s). (**E**) First image of the arena taken by the camera (beginning of the same light stimulus). (**F**) Sixth image taken by the camera (end of the same light stimulus) with *L. ingenua* individuals congregating on the bright (right) side of the arena. (**G**,**H**) Thresholded image of (**E**) and (**F**) with yellow dashed and solid lines representing the arena midperpendicular and the horizontal component of the centroid of the white pixels, respectively. Centroid bias Δ*x* is shown by horizontal yellow arrows (**G**: Δ*x* = − 0.295 cm; **H**: Δ*x* = 4.824 cm).

The experimental setup was placed in a darkened container (Fig. 1A–C). The light source was a custom-built, LED-based device being able to produce preprogrammed quasi-monochromatic light stimuli of 11 different wavelengths and the light intensity of these stimuli was variable over 3.9–4.7 log intensity units, depending on the applied wavelength. Light intensity was variable with the combination of dimming with pulse width modulation (PWM) and applying a neutral density filter (Fig. 1B, C). A circular, homogeneously illuminating area (diameter = 6 cm) provided light stimuli and available peak wavelengths (± half bandwidth) were 368 nm (± 8.2 nm), 402 nm (± 6.1 nm), 423 nm (± 5.8 nm), 446 nm (± 8.5 nm), 462 nm (± 10.8 nm), 506 nm (± 12.5 nm), 520 nm (± 13.5 nm), 557 nm (± 16.0 nm), 596 nm (± 7.2 nm), 621 nm (± 6.9 nm) and 637 nm (± 8.1 nm). The detailed description of the light source has been given elsewhere^36^.

The arena was a horizontally laid, rectangular (length = 150 mm, width = 22 mm), 3 mm deep depression in a fiberboard piece lined with black cardboard and covered by two microscope slides. The illuminating surface of the light source faced downward to the arena centre from a distance of 19 cm with its optical axis enclosing 14° with the horizontal (Fig. 1C). The arena was illuminated by 6 pieces of elemental sections of a 940 nm SMD5050 infrared LED strip and a Genius WideCam F100 webcam modified for infrared vision was aimed to the arena from above (Fig. 1A–C). The infrared background illumination enabled the observation of insects while they sensed no light.

The method for measuring the action spectrum of phototaxis of *L. ingenua* was similar as described in a recent study^36^. Different light stimuli were presented to the test insects randomly moving in darkness, and their attraction to light was quantified. At the beginning of each trial, 30–44 fungus gnats were placed in the arena and the microscope-slide-covering was quickly applied. After 30 minutes of dark adaptation, a series of 30-s-long light stimuli separated by 180-s-long dark periods were presented to the insects. Stimuli of 4–5 different light intensities were applied at all 11 wavelengths. First, at the lowest light intensity, stimuli of different wavelengths were randomly applied. Then, all available wavelengths were tested again with 1 log unit higher light intensity, and so forth. Thus, light intensity of stimuli was increasing with time (intensity step = 1 log unit), but before increasing the stimulus intensity, all wavelengths were applied randomly. From the beginning of a given stimulus, 10 photos (8-bit grayscale JPG) were taken by the camera every 6 s, thus the 1st and 6th photo corresponded to the beginning and to the end of the stimulus. The camera recorded images with 15 frames per second, thus the shutter speed for the obtained images was approximately 67 ms. As we experienced very high photosensitivity in *L. ingenua*, to extend the previously mentioned 3.9–4.7 log intensity units wide dynamic range of the light source, trials were also performed with additional, permanently attached 2 or 4 pieces of Lee 299 neutral density filters (LEE Filters, Andover, UK) [for transmission spectrum see Egri^36^]. Before each trial the live view of the fixed webcam in a custom-written software facilitated to return the arena to a standardized place. Two horizontal and two vertical guidelines were displayed on the live webcam image to which the arena edges were aligned each time. Altogether 50 trials were performed and a total number of 1834 *L. ingenua* were tested. To obtain the photon flux sensed by the test insects, wavelength dependent transmission spectrum of the microscope-slide-covering of the arena was taken into account, as well as the Fresnel’s equations applying to the air to glass and glass to air interface. Photon flux of stimuli (at the arena centre) for the test insects varied between 7.51·10^2^ and 1.53·10^13^ photons/cm^2^/s, but the applied intensity ranges per wavelength were different. Experiments were carried out at 23–26 °C and 50–55% relative humidity.

Attraction of *L. ingenua* to a given stimulus was quantified by calculating the centroid of the insects relative to the arena centre (centroid bias) at the beginning (Fig. 1G) and end of the stimulus (Fig. 1H). First of all, to obtain a static background image of the arena, the median image of the 10 images corresponding to the stimulus was calculated (Fig. 1D). The median image represented all stationary objects (e.g. arena walls, arena floor, accidental immobile insect), and this image was subtracted from a given image to be evaluated (Fig. 1E, F) and after subtraction, negative pixel values were multiplied by − 1. The resultant image was thresholded with the value of 16 (Fig. 1G, H): If a given pixel value was greater than 16, the pixel was set to white, otherwise to black. Finally, the centroid of the white pixels and its distance Δ*x* from the arena midperpendicular was calculated (centroid bias) in centimetres (Fig. 1G, H). Centroid bias was obtained for the beginning (1st image) (Fig. 1E) and end (6th image) (Fig. 1F) of each stimuli. With this method only actively moving insects contributed to the calculation of Δ*x*, because immobile insects appeared on the median image as being part of the static background.

Calculation of the action spectrum of phototaxis was the same as described by Egri et al.^36^. In short, for each wavelength, all centroid biases obtained for the end of the stimuli were plotted against log stimulus photon flux and sigmoid exposure-response curves were fitted to the data. Mean function value at the inflection points of these 11 fitted curves was considered as the critical response criterion (Δ*x*_c_ = 1.7 cm). Action spectrum of phototaxis was obtained by calculating the reciprocals of photon flux values needed for eliciting this standard response criterion. To quantify the sensitivity of the action spectrum to the criterion response value, the same calculation was performed for 20 additional values in the Δ*x*_c_ ± 0.7 cm range. All 21 action spectra were then averaged, for each data point 95% confidence intervals were calculated, and the resulting action spectrum was normalized with the maximal value.

### Behavioural experiment 2

**Figure 2:**
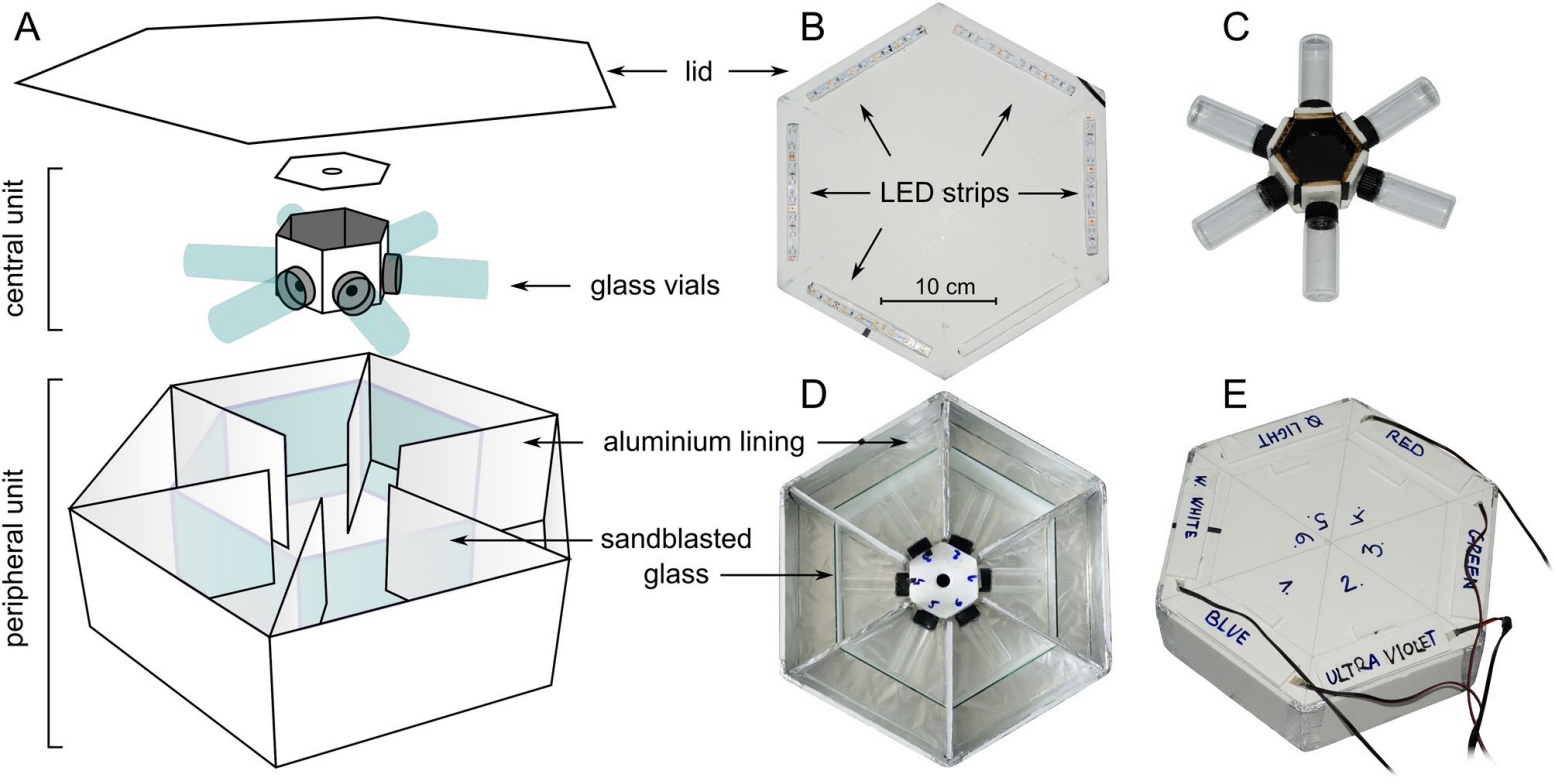
Experimental setup of behavioural experiment 2. (**A**) Exploded view of the hexagonal choice-box. (**B**) Bottom of the lid of the choice-box with the exchangeable LED strips. (**C**) Central unit with the glass vials. (**D**) Peripheral unit with the central unit inside. (**E**) Photo of the choice box with the closed lid.

In this experiment, attraction of *Lycoriella* spp. to different wavelengths of simultaneously present and equally bright light stimuli was tested in a hexagonal choice-box (Fig. 2) which was inspired by previous studies^27,37^. The choice-box was composed of a central and a peripheral unit and a lid (Fig. 2A). The central unit was a hexagonal prism (edge of base = 40 mm, height = 40 mm) with six glass vials (diameter = 27 mm, length = 70 mm) attached to each side (Fig. 2A, C). The interior of the prism was lined with black cardboard and holes (diameter = 5 mm) ensured passages for the test insects between the prism and the vials (Fig. 2A). The peripheral unit was also a hexagonal prism (edge of base = 185 mm, height = 100 mm) with its interior lined with aluminium foil and separated into six chambers (Fig. 2A, D). The central unit could be placed into the peripheral unit in such a way that each vial entered into its own chamber and the neighbouring vials could not be seen from a given vial (Fig. 2D). On the hexagonal lid of the choice-box, different LED strip sections (UV, blue, green, red or white) could be attached to illuminate the interior of the chambers from above (Fig. 2B, E). These LED strips were arbitrarily exchangeable with each other and the peak wavelengths (± half bandwidth) of the UV, blue, green and red LED strips were 398 nm (± 6.4 nm), 447 nm (± 11.3 nm), 512 nm (± 16.7 nm) and 631 nm (± 8.6 nm), respectively. The white LED strip was a 3000K warm white (WW) one. Normalized emission spectra of these LED strips are shown in Supplementary Figure S2. In each chamber a piece of vertical sandblasted glass was inserted between the vial and the illuminated end of the chamber (Fig. 2A, D). In the assembled choice-box each vial in its own chamber was homogeneously illuminated when the chamber was lit by an LED strip. Thus, viewed from the middle of the central unit, the holes (passages to the vials) as illuminating spots represented the light stimuli for the test insects. The light intensity of each LED strip was adjusted with pulse width modulation (*f*_PWM_ = 750 Hz) to a photon flux of 2.7·10^13^ photons/cm^2^/s (± 6.9%) (≈ brightness of a heavily overcast sky) measured at the passage entrances to the vials with a radiometrically calibrated Ocean Optics STS-VIS spectrometer (Ocean Optics, Largo, USA).

**Figure 3:**
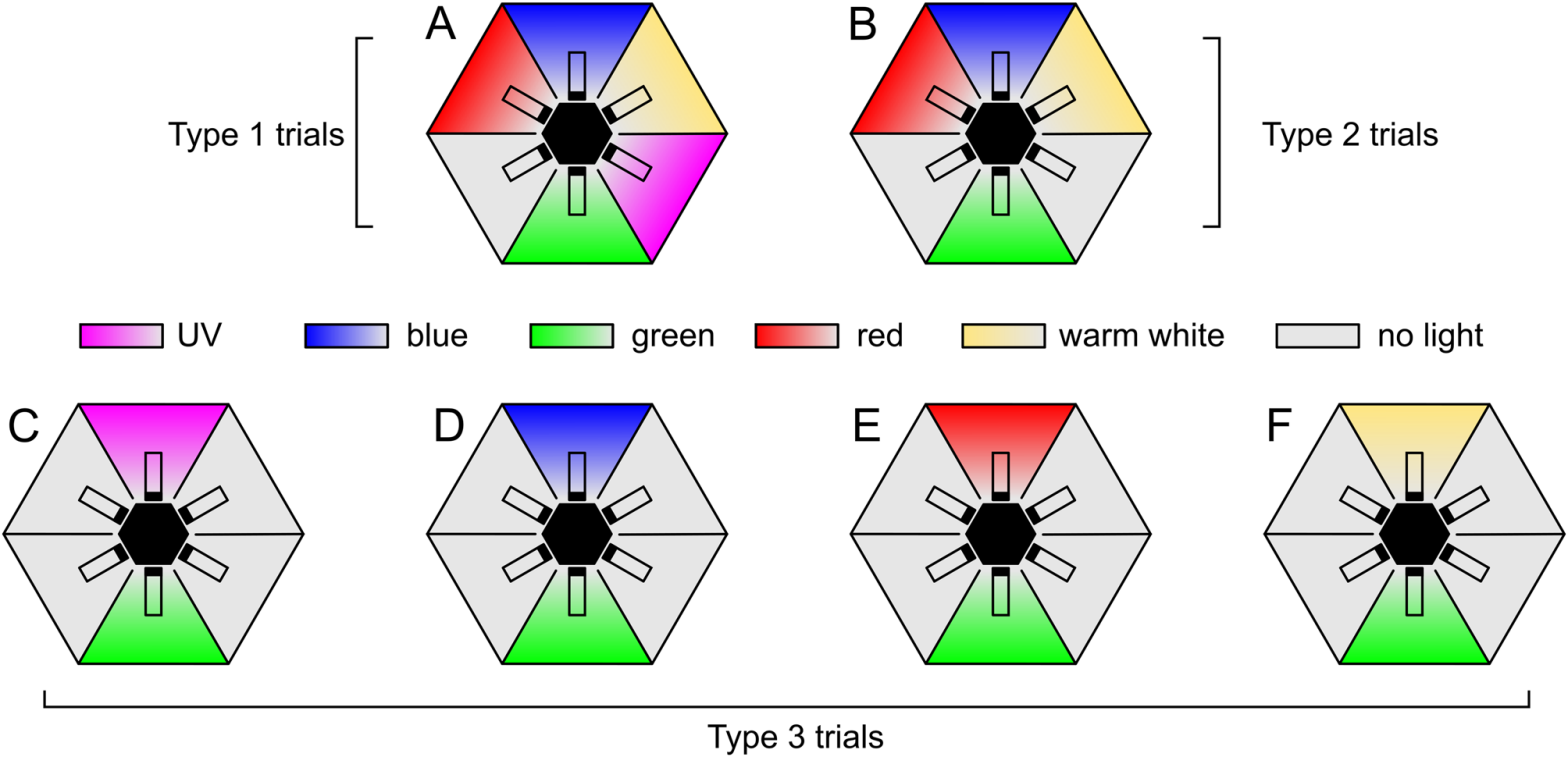
Arrangement of stimuli in the different trial types in behavioural experiment 2. (**A**) Type 1 trials (*N*_1_ = 1000). (**B**) Type 2 trials (*N*_2_ = 1000). (**C**–**F**) Type 3 trials (*N*_3_ = 4 × 500 = 2000). Arrangement of stimuli was randomized in each trial, but in type 3 trials the two applied light stimuli were always facing each other.

Trials were performed in Ócsa at the previously mentioned mushroom farm inside a production unit where *Lycoriella* spp. were abundant. Fungus gnats were collected with an aspirator and were tested immediately after collection. After setting the desired stimulus combination (LED strip arrangement) in the choice-box, 50 dark-adapted fungus gnats were placed into the middle hexagonal prism of the central unit. After 45 min the numbers of responding insects were registered for all chambers/vials of the choice-box. Figure 3 summarizes the three types of trials performed on 5 experimental days. In type 1 trials, 5 chambers of the choice-box were illuminated by the UV, blue, green, red and WW LEDs, while the sixth chamber was unexposed (Fig. 3A). Arrangement of stimuli was randomized for each trial, number of performed trials was 20, thus *N*_1_ = 1000 individuals were tested. Type 2 trials were identical with type 1 trials with the only exception that UV stimulus was excluded, thus two unexposed control chambers were present (Fig. 3B) (*N*_2 _= 1000 individuals). In type 3 trials, preference of fungus gnats to all kinds of available non-green stimuli (UV, blue, red, WW) versus green was tested in separate experiments. Light stimuli were always facing each other in the choice-box while the other four chambers were unexposed (Fig. 3C–F). Each stimulus pair was tested in 10 trials, thus *N*_3 _= 4 (stimulus pairs) × 10 (trials) × 50 (individuals) = 2000 specimens were tested in type 3 trials.

To estimate the species composition in the production unit, on each experimental day, 100 fungus gnats were collected from random locations in the unit and were later identified^3^.

### Statistics

For each tested wavelength, spectral sensitivity of female and male *L. ingenua* eye preparations were compared with Mann-Whitney U-test. In behavioural experiment 1, to test whether a stimulus (fixed wavelength and photon flux) elicited significant attraction, the distribution of Δ*x* centroid bias values at the beginning and at the end of the given stimuli were compared with Mann-Whitney U-test. To reduce the chance of false negative results, no p-value adjustments were made during data analyses^38^. In behavioural experiment 2, differences between the numbers of individuals attracted by the different chambers of the choice-box was compared with a one-way ANOVA model with the stimulus wavelength/type considered as a fixed factor. Normality of residuals was verified by Shapiro-Wilk’s test (Supplementary Table 1). To reveal statistically homogeneous groups in attraction data to the different stimuli/chambers in trial types 1 and 2 (Fig. 3A, B), Games-Howell post-hoc test was performed, because Levene’s test failed to verify homogeneity of variances. In the case of type 3 trials, based on Levene’s test results, Tukey’s post-hoc tests were performed (Fig. 3D–F), except for the green-UV stimulus arrangement (Fig. 3C), which was examined with Games-Howell post-hoc test. Since in type 3 trials all of the chambers were accessible for the fungus gnats, the two light stimuli used in these trials were also compared with the four empty chambers during data analyses. Non-responding specimens were excluded from statistical analyses.

Statistical tests were performed with the R statistical package v3.6.0^39^ and the software IBM SPSS version 22.

### Ethical approval and informed consent

No permission, licence or approval was needed for our study.

## Results

### Electroretinography measurements

**Figure 4:**
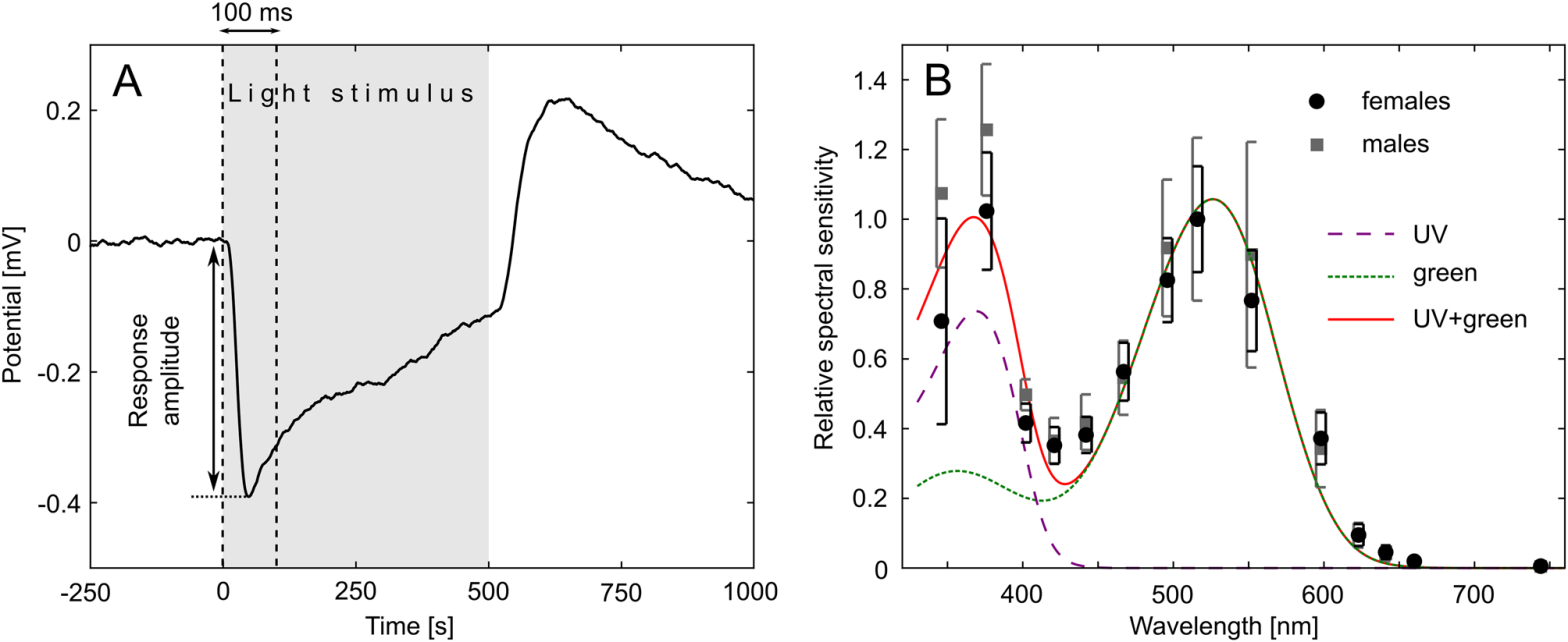
Electroretinogram of *L. ingenua*. (**A**) Time course of a typical receptor potential measured from a female *L. ingenua* eye preparation. Grey region represents the 500-ms-long light stimulus, and the pair of vertical dashed lines show the 100-ms-long period where the response amplitude was determined. (**B**) Mean relative spectral sensitivity of 12 female and 4 male *L. ingenua* eye preparations with vertical bars denoting SD. The red continuous curve shows the fitted sum of two A1-based pigment templates^41^ with peak wavelengths of λ_max,UV_ = 370.1 nm (dashed purple curve) and λ_max,G_ = 526.3 nm (dotted green curve), respectively. Curve fitting was performed on the pooled data of females and males.

Figure 4A shows the time course of a typical receptor response measurement. It is clear that the response magnitude is remarkably lower than the 10–20 mV magnitude of typical ERG signals, but electrode type and input impedance of the amplifier can strongly influence the magnitude and shape of the measured response^40^. As shown in Fig. 4B, the mean spectral sensitivity of all measured female and male *L. ingenua* eye preparations is bimodal with two maxima in the UV and green spectral regions. Even though UV-sensitivity of males seems to be higher than that of females, according to Mann–Whitney U tests, they did not differ significantly at all wavelengths. Therefore, data for females and males were pooled together in further evaluation. Assuming that two types of visual pigments dominate in the eyes, the sum of two A1-based visual pigment templates were fitted on the data. Mathematical form of the templates was obtained from eqns. (1)–(5) in Govardovskii et al.^41^. Since each template had 2 free parameters (λ_max_ and a constant parameter allowing to scale one template relative to the other), the sum of two A1-based templates had 4 parameters. Parameters giving the best fit to the measured spectral sensitivity were calculated with GNU Octave 5.2.0. For the peak wavelengths of the fitted UV and green visual templates λ_max,UV_ = 370.1 nm and λ_max,G_ = 526.3 nm were obtained, respectively (Fig. 4B).

### Behavioural experiment 1

**Figure 5:**
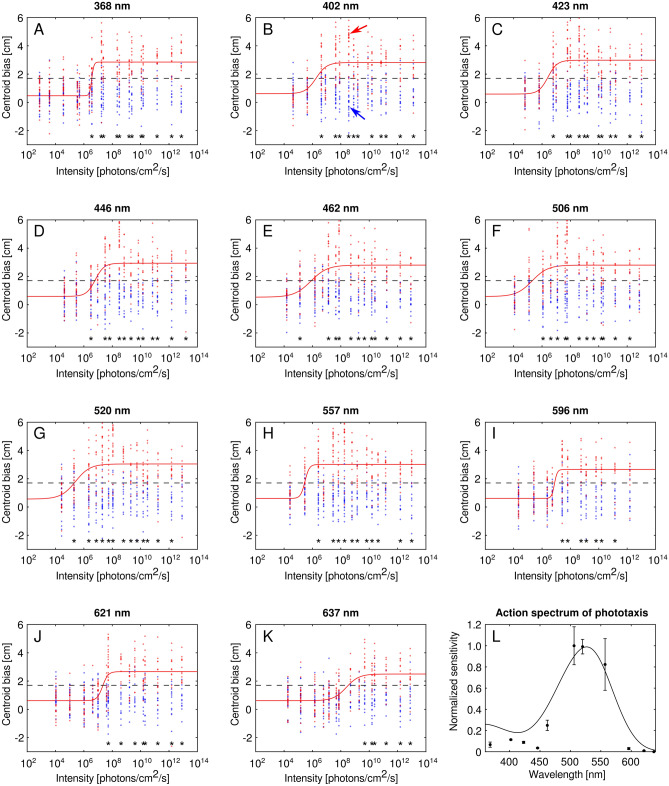
Phototactic responses of *L. ingenua* in behavioural experiment 1. (**A**–**K**) Responses with fitted sigmoid exposure-response curves at 11 wavelengths. Blue and red dots represent the centroid bias values (Δ*x*) measured at the beginning and end of the applied light stimuli. Sigmoid exposure-response curves were fitted to the Δ*x* values obtained for the end of the stimuli (red dots). Dashed lines show the critical response criterion (Δ*x*_c_ = 17.0 mm), and asterisks indicate whether distribution of the Δ*x* values at the end of a light stimulus (red dots for a given light intensity) significantly differ from the corresponding distribution of Δ*x* values measured at the beginning of the same stimulus (blue dots for the same light intensity) at *α* = 0.005 significance level. Red and blue arrows in (**B**) show Δ*x* values corresponding to Fig. 1E, G and 1F, H, respectively. (**L**) Calculated action spectrum of phototaxis with error bars denoting 95% confidence intervals. Curve shows a fitted A1-based pigment template with a λ_max_ of 526.6 nm^41^.

Results of behavioural experiment 1 are displayed in Fig. 5. In Fig. 5A–K, centroid bias values (Δ*x*) with fitted sigmoid exposure-response curves are plotted against photon flux of the light stimuli at all 11 wavelengths. Blue and red dots show Δ*x* values measured at the beginning and end of light stimuli, thus evaluation of one stimulus resulted in a pair of a blue and a red dot. Significant differences (*α* = 0.005) revealed by Mann–Whitney U test performed between the distribution of the Δ*x* values measured at the beginning and end of the stimulus for a given wavelength and photon flux are shown by asterisks. It is clear for all wavelengths that low light intensities elicited no significant reaction, but as stimulus intensity increases the attraction of fungus gnats to light becomes significant.

Figure 5L shows the calculated action spectrum of phototaxis of *L. ingenua* with vertical bars denoting 95% confidence intervals. The action spectrum has one major peak in the green spectral range and a ten times lower minor peak in the UV. An A1-based pigment template^41^ was fitted on the data and the obtained peak wavelength was λ_max_ = 526.6 nm.

### Behavioural experiment 2

**Figure 6:**
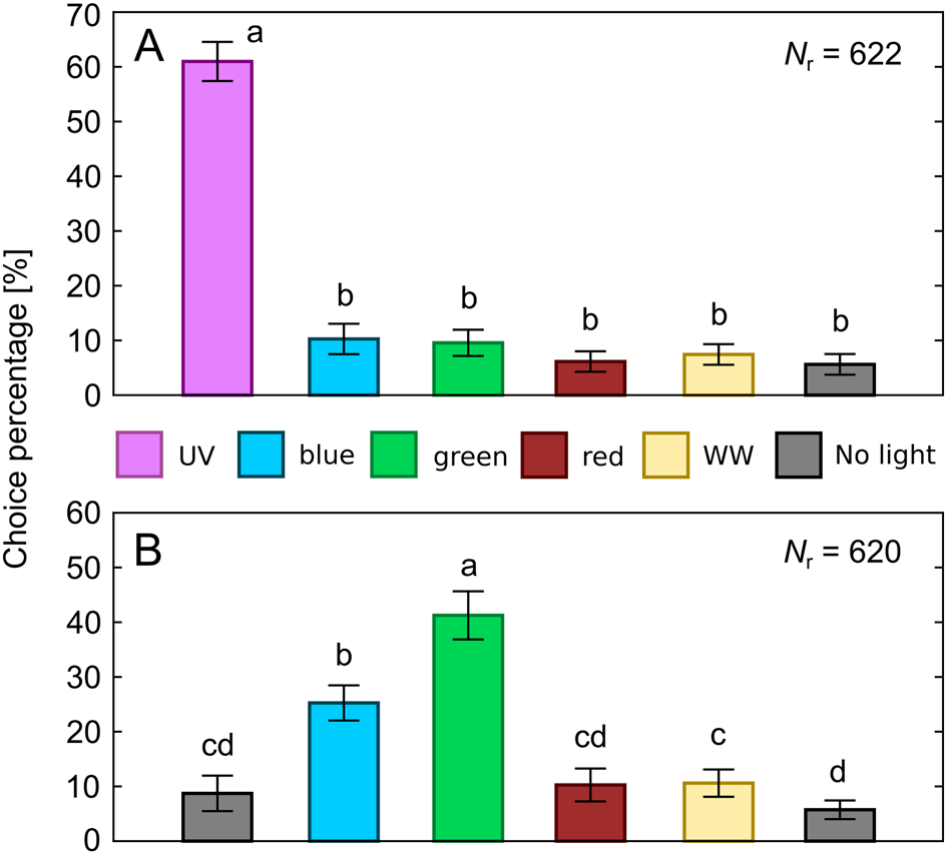
Choice percentages for the different chambers of the choice-box in trial types 1-2 in behavioural experiment 2. (**A**) Results for type 1 trials (Fig. 3A) with vertical bars denoting SD. Lowercase letters indicate statistically homogeneous groups at α = 0.05 significance level revealed by Tukey’s post-hoc test. *N*_r_ is the number of respondent fungus gnat individuals out of the total number of 1000 tested specimens. (**B**) Same as A, for type 2 trials (Fig. 3B). Total number of trials was 20 in case of both type 1 and type 2 trials.

As shown in Fig. 6A, in type 1 trials, when all available 5 LED strips plus an unexposed control chamber was present (Fig. 3A), the majority of the 622 choice-making fungus gnats chose the UV-lit chamber (60.98%). The blue-, green-, red-, and white-lit chambers and the control chamber attracted 10.27%, 9.56%, 6.14%, 7.43% and 5.62% of the respondent individuals, respectively. According to the one-way ANOVA model, significant differences were detected in these responses (*F*(5, 114) = 308.99, *p *< 0.001) and Games–Howell post-hoc test revealed that the UV light source attracted significantly more gnats than each of the other stimuli (*p *< 0.001 in each pairwise comparison with UV data).

In type 2 trials (Fig. 3B), 620 individuals made choice and the most attractive stimulus was the green (41.26% of respondents), followed by the blue (25.26% of respondents). The red, white and the two unexposed chambers attracted 10.26%, 10.60%, 8.72% and 5.73% of the respondents, respectively (Fig. 6B). The one-way ANOVA model revealed significant differences in the catch numbers of the chambers (*F*(5, 114) = 77.27, *p* < 0.001). According to Games–Howell post-hoc test, the green stimulus was significantly more attractive than any other stimuli (*p* < 0.001 in each pairwise comparison with green data). More details about statistically homogeneous groups are indicated by lowercase letters in Fig. 6B.

**Figure 7:**
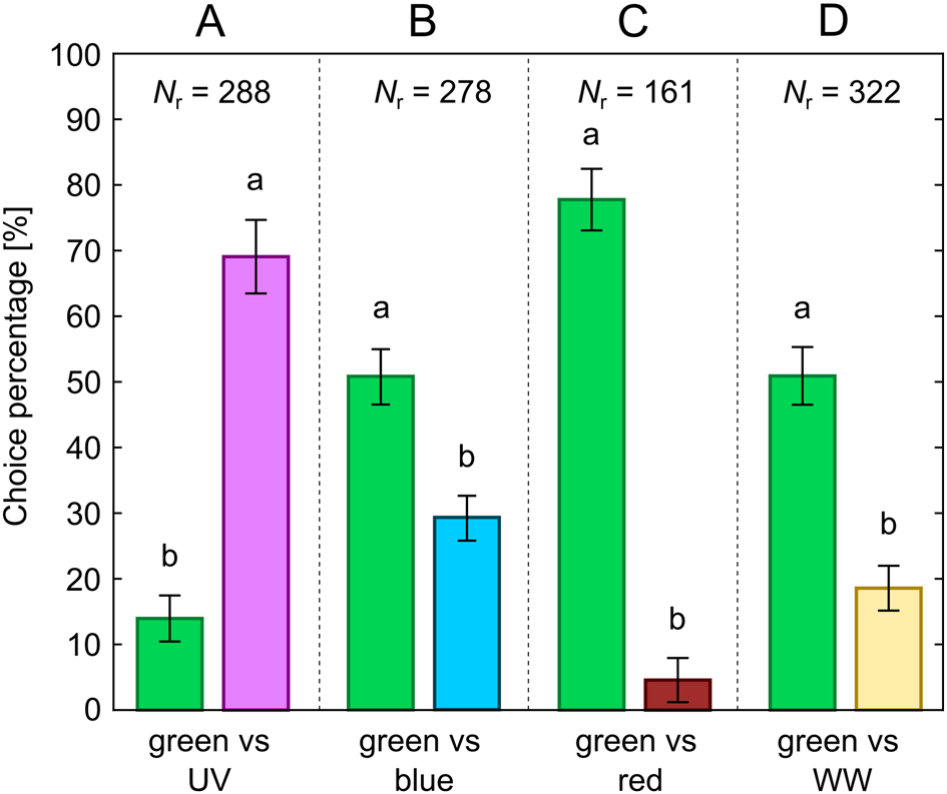
Percentage of choices of fungus gnats for the four different stimulus pairs in type 3 trials in behavioural experiment 2. (**A**) Results for the green-UV stimulus pair (Fig. 3C) with vertical bars denoting SD. Lowercase letters indicate statistically homogeneous groups at α = 0.05 significance level revealed by Games-Howell post-hoc test. Choice percentages for the four unexposed chambers are not displayed. *N*_r_ is the number of respondent fungus gnat individuals out of the total number of 500 tested specimens. Total number of trials was 10. (**B**) Same as A, for the green-blue stimulus pair (Fig. 3D) but with Tukey’s post-hoc test. (**C**) Same as (**B**), for the green-red stimulus pair (Fig. 3E). (**D**) Same as (**B**), for the green-WW stimulus pair (Fig. 3F).

Figure 7 shows the results of type 3 trials, where preference for a single light stimulus (UV, blue, red and WW) was tested against the green stimulus with the remaining four chambers being unexposed (Fig. 3C–F). Choice percentages are only shown for the given stimulus pair, but statistical tests involved the other four unexposed chambers also. Total number of respondent individuals (*N*_r_) is displayed for all stimulus configurations (top of Fig. 7). In all cases, significant differences were detected by the one-way ANOVA model (green vs UV: *F*(5, 54) = 249.90, *p* < 0.001; green vs blue: *F*(5, 54) = 181.58, *p *< 0.001; green vs red: *F*(5, 54) = 295.78, *p *< 0.001; green vs WW: *F*(5, 54) = 131.01, *p* < 0.001). According to post-hoc tests, significant difference emerged between the attraction to the green and the other stimulus (*p* < 0.05 for all type 3 trials) and catch numbers of the unexposed four chambers were statistically similar (*p *> 0.05 for all type 3 trials). Except for the UV-green stimulus pair (Fig. 7A), the green stimulus was more attractive than the other stimulus (Fig. 7B–D).

Identification of the 500 fungus gnats collected for determining the species composition in the production unit revealed that 76%, 19% and 5% of the individuals were *L. ingenua*, *L. agraria* and other *Lycoriella* spp., respectively. Consequently, the results of behavioural experiment 2 are dominated by the responses of *L. ingenua* individuals.

## Discussion

Until now, the only fungus gnat examined with ERG was the bioluminescent New Zealand glowworm *Arachnocampa luminosa* (Skuse, 1891) with eye spectral sensitivity adapted to the emission spectrum of the bioluminescence of the individuals^42^. The subject of our present work, *L. ingenua* is not that special as *A. luminosa*, but its significance as a crop pest is infinitely superior.

According to our ERG measurements, *L. ingenua* has two sensitivity maxima, one in the UV and another in the green spectral range. Only from dark-adapted ERG recordings one cannot unambiguously identify different photoreceptor types, although distinct sensitivity peaks may indicate that more than one photoreceptors are present^42^. The fact that phototaxis was far the strongest for green and UV quasi monochromatic light in behavioural experiments 1 and 2, respectively, may also indicate that the presence of the peaks in the spectral sensitivity of the compound eye (Fig. 4B) are caused by distinct photoreceptor types. The reason for the different attraction to light as a function of wavelength lies in the differences between the two setups.

There were three essential differences between behavioural experiments 1 and 2. Firstly, in behavioural experiment 1, only one stimulus was present at a time, while in behavioural experiment 2, stimuli were simultaneously present. Secondly, the results of behavioural experiment 1 (Fig. 5) and 2 (Figs. 6, 7) are primarily valid for different light intensity ranges. In behavioural experiment 1, typical minimal photon flux values being able to elicit significant attraction depended on wavelength, but were in the 10^5^–10^9^ photons/cm^2^/s range even in the case of the least attractive red wavelengths. These photon flux values are excessively lower than that of the stimuli used in behavioural experiment 2 (~10^13^ photons/cm^2^/s). This is not especially surprising, because methodology applied in behavioural experiment 1 is based on the determination of the minimal light intensity needed for a given phototactic reaction (critical response criterion Δ*x*_c_), and photon flux of the stimuli in behavioural experiment 2 was arbitrarily chosen. The third important difference between the behavioural experiments was related to the test insects. Behavioural experiment 1 was made exclusively with *L. ingenua*, while in behavioural experiment 2, the proportion of this species was 76%, however, the rest 24% could not account for the high UV preference (Figs. 6A, 7A) alone.

Thus, it seems that spectral sensitivity of phototaxis in *L. ingenua* depends on light intensity.

Explanation for our results may lie in the ocelli, which are simple eyes usually present in winged adult insects^43^. Ocelli are extremely underfocused visual organs and mostly used to stabilize flight by blurry but rapidly perceiving the intensity pattern formed by the dark ground and the brighter sky^22^. Although ocelli thought to be unable to mediate phototactic reactions alone^44^, light reaching exclusively the ocelli can elicit phototaxis from insects after all^45,46^. Because primarily green-sensitive ocelli are not uncommon among insects^47–49^, and the light sensitivity of insect ocelli can be several orders of magnitude higher than that of the compound eyes^50^, light perception through ocelli could have been dominated in behavioural experiment 1 resulting in the action spectrum of phototaxis with λ_max_ = 526.6 nm (Fig. 5L). Green-sensitive ocelli is a feature of mainly nocturnal insects^47^, because night-light has very little UV content, especially under forest canopies^22^. Among fungus gnats, both diurnality and nocturnality has already been demonstrated^51–53^, but as far as we know, circadian activity of *L. ingenua* has not been studied yet. In behavioural experiment 2, the higher stimulus intensities could account for the mainly UV-dominated attraction (Fig. 6A) which result is in accordance with the finding of Stukenberg et al.^29^ who used practically the same light intensities for quasi monochromatic stimuli in choice experiments with the black fungus gnat *B. difformis*, being another notorious pest in mushroom cultivation. When UV stimulus was not present in behavioural experiment 2 (Figs. 6B, 7B–D), the green stimulus was the most attractive which is also in accordance with previous studies^28^.

On the other hand, the peak of the action spectrum of phototaxis (Fig. 5L) overlaps extremely well with the green sensitivity peak in the spectral sensitivity of the compound eye (λ_max,G_ = 526.3 nm, Fig. 4B), which may suggest that the compound eye could still play significant role in behavioural experiment 1. It is also important to mention that in behavioural experiment 2, only four quasi-monochromatic stimuli were applied and the shortest-wavelength stimulus was the UV (398 nm) one. Consequently, the wavelength of maximal attraction cannot be estimated as precisely as could be done for behavioural experiment 1 (Fig. 5L), but based on the ERG results it is expected around 370 nm (Fig. 4B). However, independent of the underlying mechanisms causing the light intensity dependent reactions, our experiments and ERG recordings highlight the importance of both UV (~ 370–398 nm) and green (~ 526 nm) light, or even their combination for creating visually attractive monitoring or trapping tools for *L. ingenua* at mushroom growing facilities.

In general, the spectral sensitivity obtained from ERG recordings is very informative, but the action spectrum of phototaxis is not necessarily similar in shape. Thus performing behavioural experiments are essential in the process of light trap development. The most important message of our paper is that UV and green spectral ranges attract *L. ingenua* individuals with the highest efficiency and future research should investigate the effectiveness of trap prototypes in combining these spectral regions. Based on our results, we would also like to emphasize that besides the wavelength composition of a light stimulus, light intensity is also a very important parameter, which should not be disregarded when light trapping of insects is the aim.

## Supplementary information

Supplementary Information.

## Data Availability

Our paper has a supplementary table and two supplementary figures.

## References

[1] Binns ES (1981). Fungus gnats (Diptera: Mycetophilidae/Sciaridae) and the role of mycophagy in soil: A review. Rev. Ecol. Biol. Sol..

[2] Fletcher, J. T. & Gaze, R. H. Pests. In *Mushroom Pest and Disease Control: A Color Handbook* 140–165 (Manson Publishing Ltd, 2008).

[3] Menzel, F. & Mohrig, W. *Revision der paläarktischen Trauermücken (Diptera, Sciaridae)*. (Ampyx-Verlag, 2000).

[4] Mead, F. W. & Fasulo, T. R. Darkwinged Fungus Gnats, *Bradysia* spp. (Insecta: Diptera: Sciaridae). *Entomology and Nematology Department Series of Florida University. UF/IFAS Extension* **14**, 1–3 (2001).

[5] Cloyd RA (2015). Ecology of fungus gnats (*Bradysia* spp.) in greenhouse production systems associated with disease-interactions and alternative management strategies. Insects.

[6] Mohrig, W. & Menzel, F. Sciaridae (Black Fungus Gnats). in *Manual of Central American Diptera* (eds. Brown, B. V. et al.) vol. 1 279–292 (NRC Research Press, 2009).

[7] Taylor GJ, Hall SA, Gren JA, Baird E (2020). Exploring the visual world of fossilized and modern fungus gnat eyes (Diptera: Keroplatidae) with X-ray microtomography. J. R. Soc. Interface..

[8] White, P. F. Pest and Pesticides. in *The Biology and Technology of the Cultivated Mushroom* (eds. Flegg, P. B., Spencer, D. M. & Wood, D. A.) 279–294 (Wiley, 1985).

[9] Shamshad A (2010). The development of integrated pest management for the control of mushroom sciarid flies, *Lycoriella ingenua* (Dufour) and *Bradysia ocellaris* (Comstock), in cultivated mushrooms: IPM for control of sciarid flies in mushrooms. Pest. Manag. Sci..

[10] Andreadis SS, Cloonan KR, Myrick AJ, Chen H, Baker TC (2015). Isolation of a female-emitted sex pheromone component of the fungus gnat, *Lycoriella ingenua*, attractive to males. J. Chem. Ecol..

[11] Hussey NW, Gurney B (1968). Biology and control of the sciarid *Lycoriella auripila* Winn. (Diptera: Lycoriidae) in mushroom culture. Ann. Appl. Biol..

[12] White PF (1986). The effect of sciarid larvae (*Lycoriella auripila*) on cropping of the cultivated mushroom (*Agaricus bisporus*). Ann. Appl. Biol..

[13] Shamshad A, Clift AD, Mansfield S (2009). The effect of tibia morphology on vector competency of mushroom sciarid flies. J. Appl. Entomol..

[14] Chang, S. T. & Miles, P. G. Insect diseases. in *Mushrooms: Cultivation, Nutritional Value, Medicinal Effect, and Environmental Impact* 179–185 (CRC Press, 2004).

[15] O’Connor L, Keil CB (2005). Mushroom host Influence on *Lycoriella mali* (Diptera: Sciaridae) life cycle. J. Econ. Entomol..

[16] Badshah K (2020). Management of *Lycoriella ingenua* (Diptera: Sciaridae) on oyster mushroom (*Pleurotus ostreatus*) through different botanicals. Int. J. Trop. Insect Sci..

[17] Cloonan KR, Andreadis SS, Baker TC (2016). Attraction of female fungus gnats, *Lycoriella ingenua,* to mushroom-growing substrates and the green mold *Trichoderma aggressivum*. Entomol. Exp. Appl..

[18] Geösel A (2016). A termesztett csiperkegomba védelme. Növényvédelem.

[19] Grewal PS, Tomalak M, Keil CBO, Gaugler R (1993). Evaluation of a genetically selected strain of *Steinernema feltiae* against the mushroom sciarid *Lycoriella mali*. Ann. Appl. Biol..

[20] Jess S, Bingham JFW (2004). Biological control of sciarid and phorid pests of mushroom with predatory mites from the genus *Hypoaspis* (Acari: Hypoaspidae) and the entomopathogenic nematode *Steinernema feltiae*. Bull. Entomol. Res..

[21] European Commission. Report from the Commission to the European Parliament and the Council. COM(2020) 204 final. https://eur-lex.europa.eu/legal-content/EN/TXT/?uri=CELEX:52020DC0204 (2020).

[22] Cronin TW, Johnsen S, Marshall NJ, Warrant EJ (2014). Visual Ecology.

[23] Shimoda M, Honda K (2013). Insect reactions to light and its applications to pest management. Appl. Entomol. Zool..

[24] Kim M-G, Yang J-Y, Lee H-S (2013). Phototactic behavior: Repellent effects of cigarette beetle, *Lasioderma serricorne* (Coleoptera: Anobiidae), to light-emitting diodes. J. Korean Soc. Appl. Biol. Chem..

[25] Stringer I, Meyer-Rochow V (1994). Attraction of flying insects to light of different wavelengths in a Jamaican cave. Mémoires de Biospeologie.

[26] Jess S, Bingham JFW (2004). The spectral specific responses of *Lycoriella ingenua* and *Megaselia halterata* during mushroom cultivation. J. Agric. Sci..

[27] Cloyd, R. A., Dickinson, A., Larson, R. A. & Marley, K. A. Phototaxis of fungus gnat, *Bradysia* sp. nr *coprophila* (Lintner) (Diptera: Sciaridae), adults to different light intensities. *HortScience* **42**, 1217–1220 (2007).

[28] Kim HH (2014). Attract effect of mushroom flies with different wavelength of light emitting diode(LED). J. Mushroom.

[29] Stukenberg N, Ahrens N, Poehling H-M (2018). Visual orientation of the black fungus gnat, Bradysia difformis, explored using LEDs. Entomol. Exp. Appl..

[30] An L (2019). High innate preference of black substrate in the chive gnat, *Bradysia odoriphaga* (Diptera: Sciaridae). PLoS ONE.

[31] Kühne, S. & Heller, K. Sciarid fly larvae in growing media—biology, occurrence, substrate and environmental effects and biological control measures. in *Peat in Horticulture—Life in Growing Media* (ed. Schmilewski, G.) 95–102 (International Peat Society, 2010).

[32] Broadley A, Kauschke E, Mohrig W (2018). Black fungus gnats (Diptera: Sciaridae) found in association with cultivated plants and mushrooms in Australia, with notes on cosmopolitan pest species and biosecurity interceptions. Zootaxa.

[33] Egri Á, Kriska G (2019). How does the water springtail optically locate suitable habitats? Spectral sensitivity of phototaxis and polarotaxis in *Podura aquatica*. J. Exp. Biol..

[34] Land BR, Wyttenbach RA, Johnson BR (2001). Tools for physiology labs: An inexpensive high-performance amplifier and electrode for extracellular recording. J. Neurosci. Methods.

[35] DeVoe RD, de-Souza JM, Ventura DF (1997). Electrophysiological measurements of spectral sensitivities: a review. Braz. J. Med. Biol. Res..

[36] Egri Á, Farkas P, Bernáth B, Guerin PM, Fail J (2020). Spectral sensitivity of L2 biotype in the *Thrips tabaci* cryptic species complex. J. Insect Physiol..

[37] Yang EC, Lee DW, Wu WY (2003). Action spectra of phototactic responses of the flea beetle, *Phyllotreta striolata*. Physiol. Entomol..

[38] Rothman KJ (1990). No adjustments are needed for multiple comparisons. Epidemiology.

[39] R Core Team. *R: A Language and Environment for Statistical Computing*. (Foundation for Statistical Computing, 2019).

[40] Kugel M (1977). The time course of the electroretinogram of compound eyes in insects and its dependence on special recording conditions. J. Exp. Biol..

[41] Govardovskii VI, Fyhrquist N, Reuter T, Kuzmin DG, Donner K (2000). In search of the visual pigment template. Vis. Neurosci..

[42] Meyer-Rochow VB, Eguchi E (1984). Thoughts on the possible function and origin of bioluminescence in the New Zealand glowworm *Arachnocampa luminosa* (Diptera: Keroplatidae), based on electrophysiological recordings of spectral responses from the eyes of male adults. N. Z. Entomol..

[43] Land MF, Nilsson D-E (2012). Animal Eyes.

[44] Cornwell PB (1955). The functions of the ocelli of Calliphora (Diptera) and Locusta (Orthoptera). J. Exp. Biol..

[45] Lazzari CR, Reiseman CE, Insausti TC (1998). The role of the ocelli in the phototactic behaviour of the haematophagous bug *Triatoma infestans*. J. Insect Physiol..

[46] Wehrhahn C (1984). Ocellar vision and orientation in flies. Proc. R. Soc. Lond. B.

[47] Berry RP, Wcislo WT, Warrant EJ (2011). Ocellar adaptations for dim light vision in a nocturnal bee. J. Exp. Biol..

[48] Goldsmith TH, Ruck PR (1958). The spectral sensitivities of the dorsal ocelli of cockroaches and honeybees: An electrophysiological study. J. Gen. Physiol..

[49] Lall AB, Ovid Trouth C (1989). The spectral sensitivity of the ocellar system in the cricket *Gryllus firmus* (Orthoptera: Gryllidae). J. Insect Physiol..

[50] Wilson M (1978). The functional organisation of locust ocelli. J. Comp. Physiol..

[51] Duque-Buitrago CA, Alzate-Quintero NF, Tupac Otero J (2014). Nocturnal pollination by fungus gnats of the colombian endemic species, *Pleurothallis marthae* (orchidaceae: pleurothallidinae). Lankesteriana.

[52] Li HJ, He XK, Zeng AJ, Liu YJ, Jiang SR (2007). *Bradysia Odoriphaga* copulatory behavior and evidence of a female sex pheromone. J. Agric. Urban Entomol..

[53] Mochizuki K, Kawakita A (2018). Pollination by fungus gnats and associated floral characteristics in five families of the Japanese flora. Ann. Bot..

