# Supplementary material for: In search of the spectral composition of an effective light trap for the mushroom pest Lycoriella ingenua (Diptera: Sciaridae)
Source: Sci Rep. 2021 Jun 17;11:12770. doi: 10.1038/s41598-021-92230-y (PMC8211823; doi:10.1038/s41598-021-92230-y)
Supplement: Supplementary file 1 — Supplementary Information. [file 41598_2021_92230_MOESM1_ESM.pdf]

Supplementary material for

In search of the spectral composition of an effective light trap for the mushroom pest *Lycoriella ingenua* (Diptera: Sciaridae)

Sándor Kecskeméti, András Geösel, József Fail and Ádám Egri*

*Corresponding author, e-mail address: egri.adam@ecolres.hu

This document contains 1 supplementary table and 2 supplementary figures.

Table

Supplementary Table 1: Results of Shapiro-Wilk's test for verifying data normality for the trials of behavioural experiment 2.

Trial type	Statistics
Type 1 trial	$D(120) = 0.979$; $p = 0.061$
Type 2 trial	$D(120) = 0.987$; $p = 0.308$
Type 3 trial – green vs UV	$D(60) = 0.990$; $p = 0.889$
Type 3 trial – green vs blue	$D(60) = 0.974$; $p = 0.223$
Type 3 trial – green vs red	$D(60) = 0.963$; $p = 0.070$
Type 3 trial – green vs WW	$D(60) = 0.968$; $p = 0.123$

Figures

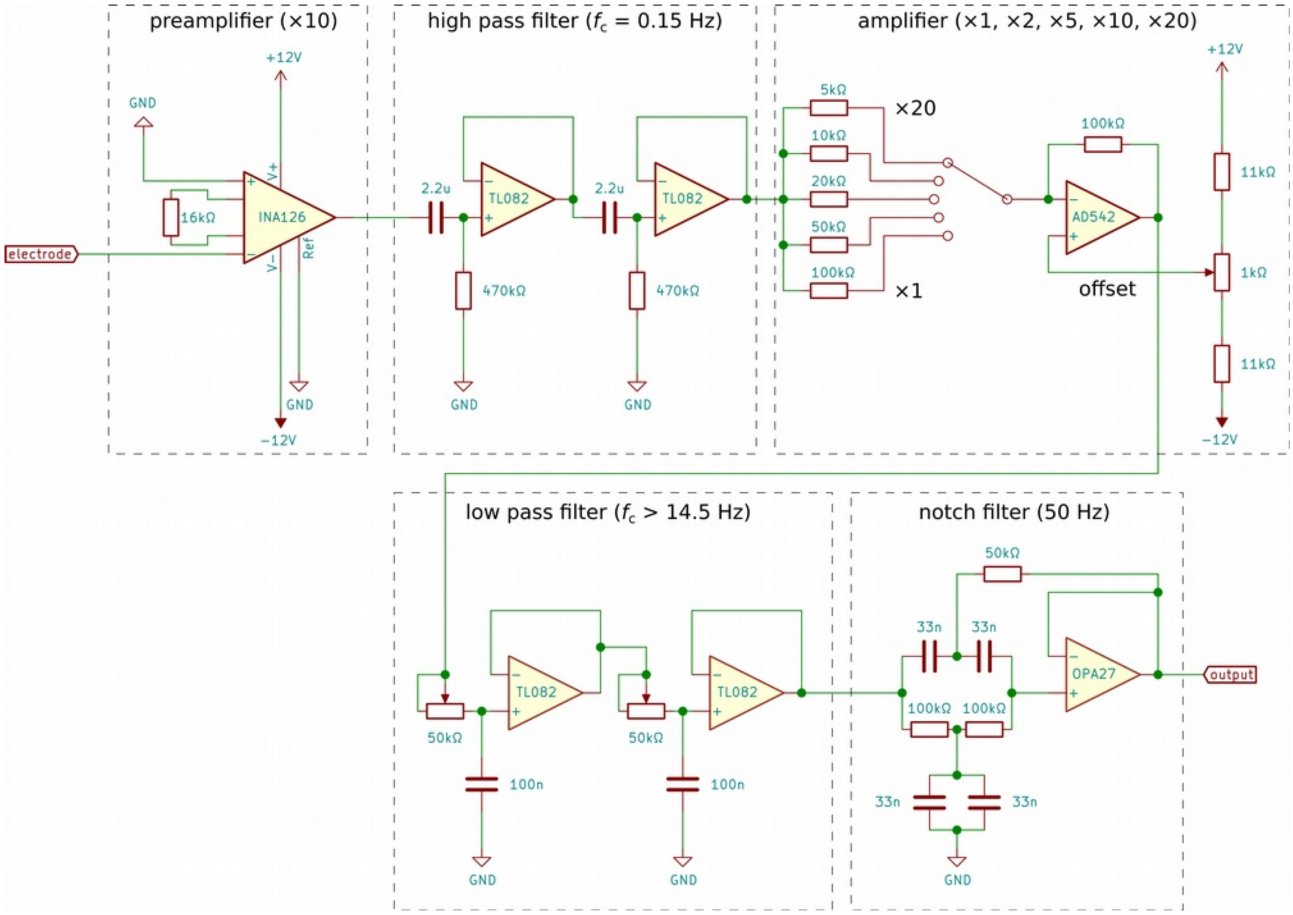

Supplementary Figure S1: Electronic circuit diagram of the amplifier used in the electroretinography measurements.

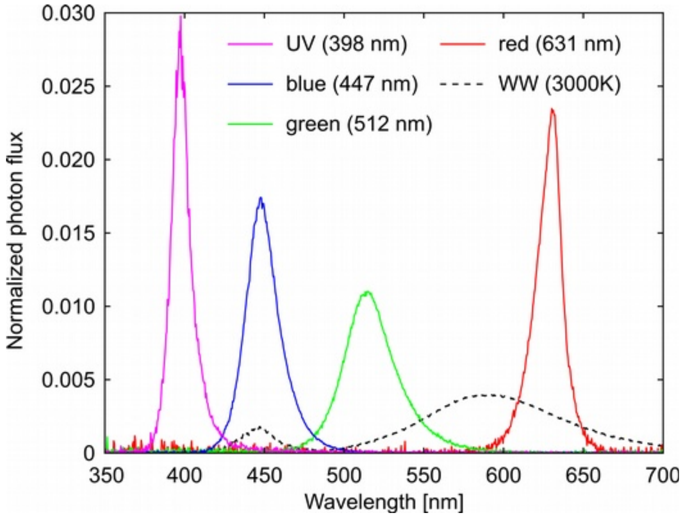

Supplementary Figure S2: Normalized emission spectra (area under each curve equals 1) of the five different LED strips used in the trials of behavioural experiment 2.
